# Comparison of Carotid Plaque Ultrasound and Computed Tomography in Patients and Ex Vivo Specimens—Agreement of Composition Analysis

**DOI:** 10.3390/jcm15020545

**Published:** 2026-01-09

**Authors:** Simon Stemmler, Martin Soschynski, Martin Czerny, Thomas Zeller, Dirk Westermann, Roland-Richard Macharzina

**Affiliations:** 1 Department of Cardiology and Angiology, University Heart Center Freiburg–Bad Krozingen, Faculty of Medicine, University of Freiburg, 79085 Freiburg im Breisgau, Germany; thomas.zeller@uniklinik-freiburg.de (T.Z.); dirk.westermann@uniklinik-freiburg.de (D.W.); roland.macharzina@uniklinik-freiburg.de (R.-R.M.); 2 Department of Anesthesiology, University Medical Center Hamburg-Eppendorf, 20251 Hamburg, Germany; 3 Department of Computer Science, University of Oxford, Oxford OX1 3QD, UK; 4 Department of Diagnostic and Interventional Radiology, University Medical Center Freiburg, Faculty of Medicine, University of Freiburg, 79085 Freiburg im Breisgau, Germany; martin.soschynski@uniklinik-freiburg.de; 5 Department of Cardiovascular Surgery, University Heart Center Freiburg–Bad Krozingen, 79085 Freiburg im Breisgau, Germany; martin.czerny@uniklinik-freiburg.de

**Keywords:** artificial intelligence, radiomics, CT, ultrasound, plaque imaging, carotid stenosis, carotid endarterectomy

## Abstract

**Background:** Carotid plaque composition is central to stroke risk, but some aspects of plaque characterization are derived from ex vivo imaging, while clinical decision-making relies on in vivo ultrasound (US) and computed tomography (CT). High correlation of clinical in vivo and ex vivo imaging is necessary when including ex vivo plaque features in artificial intelligence (AI) models, but the extent of this correlation between CT and US remains poorly understood. **Methods:** Patients undergoing carotid endarterectomy (n = 188) were enrolled. Preoperative carotid US (n = 182) and CT (n = 156) were performed. Plaque specimens from 187 patients were imaged on ex vivo CT and US. Quantitative metrics included plaque volumes, relative calcified/non-calcified volumes, HU and grayscale distributions, Agatston and calcification scores, and heterogeneity indices (coefficient of variation). Qualitative US parameters (echogenicity, juxtaluminal echolucency, discrete white areas) were visually graded. Correlation between in vivo and ex vivo imaging was assessed, and agreement was quantified for parameters with the highest correlation with Bland–Altman analysis. **Results:** CT of patients and ex vivo CT showed moderate to strong correlation for total, calcified, and non-calcified plaque volumes and whole-plaque mean HU (r = 0.55–0.79; CCC = 0.43–0.74). Agatston and calcification scores correlated strongly (r = 0.78–0.80; CCC = 0.63–0.76). In contrast, most non-calcified and heterogeneity metrics showed negligible-to-weak correlation. Correlations between in vivo and ex vivo US were substantially weaker (maximum correlation: 75th grayscale percentile r = 0.35). In vivo CT overestimated calcified volume (bias: 8.7%) and in vivo US underestimated the 75th grayscale quantile (bias: −25.5 grayscale). **Conclusions:** Quantitative CT metrics—particularly relative calcified plaque volume and calcium scores—translate reasonably well from ex vivo to in vivo imaging and represent robust candidates for radiomics and AI-based stroke risk models, even ex vivo. Ultrasound parameters show limited translational validity, underscoring the need for volumetric clinical US and discouraging the inclusion of ex vivo ultrasound features for machine learning applications.

## 1. Introduction

Carotid artery stenosis is a major cause of stroke and contributes substantially to morbidity and mortality [1]. Carotid endarterectomy (CEA) is an established treatment, yet current guidelines base revascularization decisions on the degree of stenosis and neurological symptoms, with only modest consideration of imaging features [2,3]. Growing evidence indicates that plaque composition assessed by ultrasound (US) and computed tomography (CT) can distinguish high- from low-risk lesions [4]. A recent trial questioned the benefit of CEA over the best medical treatment, further underscoring the need for improved preoperative risk stratification [5].

Advanced computational approaches, including artificial intelligence and machine learning, offer the possibility to integrate multiple CT and US features into multimodal risk models [6,7]. However, many imaging characteristics linked to cerebrovascular events originate from heterogeneous sources—often from different modalities in patients or even from ex vivo analyses of resected plaques—which complicates translation into clinical decision-making [4]. CT imaging of carotid plaque specimens has been frequently performed and has revealed multiple features of patients with symptomatic plaques. Plaque components such as calcification and lipid tissue could be identified ex vivo per Hounsfield Unit (HU) values and a calcification score was calculated with high correlation to histology [8,9]. High-resolution CT of specimens was even used to validate the identification of calcification on CT in patients [10]. US of carotid plaques accurately associated the grayscale median (GSM) with lipid-rich and fibrous components, but not with calcification [9,11]. Sophisticated US techniques, such as spectral analysis of backscattered radiofrequency signals and complex analyses of grayscale distribution within the plaque, showed high accuracy for assessing potentially vulnerable plaque components [12,13]. Multimodal imaging of carotid specimen phantoms showed good detection rates of ulcerations and surface irregularity on US and CT, but not on MRI [14]. Ex vivo imaging provides unique advantages by revealing the full potential of what clinical imaging may be able to detect, but how well these ex vivo findings correspond to in vivo patient imaging remains insufficiently understood [15]. For instance, ex vivo dual-energy CT has shown substantial improvements in plaque characterization, and intravascular US could virtually assess the histological composition of plaques [15,16,17]. The correlation of ex vivo and in vivo CT images has only been studied inconclusively in 16 patients, suggesting strong correlation with underestimation of calcification by in vivo CT [18]. Data for correlation of in vivo and ex vivo US do not exist. In most studies, plaque criteria that appear highly valuable for determining stroke risk are mentioned, but also in vivo validation is warranted; however, the translatability of ex vivo to in vivo imaging remains poorly understood. Ex vivo CT plaque analysis has indicated that calcification, lipid-rich necrotic core, and fibrous tissue can be accurately characterized, but although the translation to in vivo has been questioned, these features can still be found in contemporary imaging characterizations recommended for patients [8,9,10,15]. Similarly, ultrasound-based plaque metrics, such as grayscale median, backscatter analysis, and qualitative echogenicity grading, have been associated with symptoms from ex vivo studies [11,12,13]. Consideration of these features by AI models of radiomics necessitates high correlation of in and ex vivo imaging, which is currently unknown.

To address this gap, we conducted a comparative multimodal analysis, matching US and CT plaque component imaging in patients with ex vivo imaging of the corresponding CEA specimens using clinically applicable techniques and devices.

## 2. Materials and Methods

### 2.1. Patients, Carotid Endarterectomy, and Specimen Preparation

**Figure 1:**
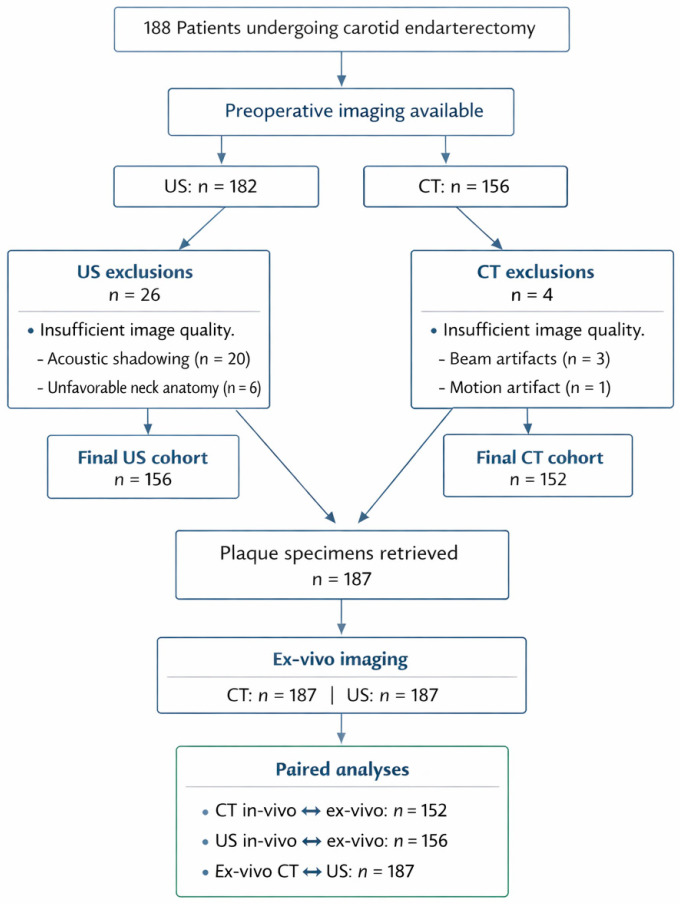
Flow chart. Patients undergoing carotid endarterectomy received preoperative in vivo computed tomography (CT) and ultrasound (US). Specimens of patients were also imaged on CT and US and imaging characteristics were compared in vivo to ex vivo.

This study followed rules of good clinical practice and the standard criteria of diagnostic imaging studies (STARD) (http://www.stard-statement.org; http://ichgcp.net/) and was approved by the ethics committee of the Albert-Ludwigs-University Freiburg (ID 439/14). A total of 188 patients with an indication for carotid endarterectomy were enrolled, of which 182 patients received carotid US (external studies: n = 6) and 156 patients received CT of the carotid plaque (magnetic resonance imaging: n = 32). Patient characteristics have been previously described [15,19]. Briefly, patients represent a cohort reflective of a tertiary cardiovascular center. Patients with insufficient image quality were excluded on US (n = 26; acoustic shadowing n = 20; unfavorable neck anatomy n = 6) and on CT (n = 4; beam hardening artifacts n = 3; motion artifacts n = 1), resulting in 156 patients for US analysis and 152 patients for CT analysis. From 187 patients, carotid plaque specimens were retrieved after CEA, and all were imaged on ex vivo US and CT (Figure 1). Clinical characteristics, preoperative neurological status, and stenosis degree were recorded. Experienced vascular surgeons performed CEA. After rinsing the specimens, they were fixed in pH-neutral 4% formaldehyde and stored for analysis.

### 2.2. Computed Tomography

CT of patients was performed as previously described [19]. In brief, imaging was performed on a 64-row CT scanner (SOMATOM Sensation Plus, Siemens, Munich, Germany) with 120 kV and 240 mAs. The degree of stenosis, calcified plaque volume, HU density, and heterogeneity (coefficient of variation (CV), standard deviation divided by average HU) as well as localization of calcification (deep/adventitial vs. superficial/luminal), number of calcification spots <1 mm, and calcification and Agatston scores were assessed [19,20].

CT of ex vivo plaque specimens was performed as previously reported [15]. In short, plaques were placed in a dual-source CT gantry (SOMATOM Force, Siemens Healthineers, Erlangen, Germany) and an ultra-high-resolution single-energy (120 kV; 500 mAs) protocol was applied. Calcification was subclassified by location, calcification spots <1 mm, calcification score, and Agatston score [15,20]. Plaque volume was analyzed for Hounsfield unit (HU) density, calcified proportion, and heterogeneity as the CV.

### 2.3. Ultrasound

Ultrasound of patients was performed using a Philips IU22 system (L9–3 MHz transducer; PHILIPS, Hamburg, Germany). B-mode settings were optimized, and the maximal luminal narrowing most representative of the entire plaque was selected while avoiding acoustic shadowing. Images were saved and analyzed on ImageJ 1.53c (National Institutes of Health, Bethesda, MD, USA) by a blinded observer. Grayscale (GS) values were normalized to the adventitial layer (reference = 190) [21]. Histograms of GS were created, excluding GS = 0 to remove blood signal. Average, median (GSM), quartiles, coefficient of variation (CV) of GS, and the share with GS < 25 or <35 were calculated. Additional plaque features—including echogenicity, juxtaluminal echolucency, and discrete white areas (DWA)—were assessed [21,22].

Plaque specimens were imaged on the same workstation. Plaques were positioned inside phantoms consisting of a polypropylene cup, 5% agarose gel, and phosphate-buffered saline next to a 15 × 10 × 10 mm bovine aortic reference sample. Gain and specimen placement were optimized. Three-dimensional images were achieved by transducer movement along the plaque and DICOM files were created. Histograms of GS values were calculated, and GS were referenced to the bovine adventitial layer. Voxel size in the z-direction was calculated. Voxels with GS = 0 were excluded. GS average, median (GSM), quartiles, CV, and the share with GS < 25 or <35 were calculated. Qualitative assessment of echogenicity, juxtaluminal echolucency, and discrete white areas (DWA) was performed. The predominant appearance across the plaque determined the criterion, except for DWA (any presence).

### 2.4. Statistics and Comparative Analysis

Agreement between in vivo and ex vivo imaging was quantified using Pearson’s correlation coefficient (r) and Lin’s concordance correlation coefficient (CCC) for continuous variables, and the Phi coefficient (Φ) for binary parameters. Correlations were considered negligible (<0.1), weak (0.1–0.39), moderate (0.4–0.69), strong (0.70–0.89), or very strong (>0.89) [23]. Both r and CCC were reported to distinguish linear association from concordance at the line of equality. For index parameters such as the coefficient of variation or relative volumes, values were expected to cluster near the line of equality; thus, the CCC was the preferred measure. For absolute parameters (e.g., HU values, absolute volumes), systematic over or underestimation between modalities was anticipated due to technical differences, making r the more informative metric [24]. For measurements with the highest correlation on US and CT, Bland–Altman analysis was performed to assess over/underestimation (bias) and 95% limits of agreement.

For CT comparisons, ex vivo CT served as the reference standard owing to its higher spatial resolution, absence of contrast-related artifacts, semi-automated HU-based volumetry, and expanded diagnostic capability. Even for parameters without methodologic superiority (e.g., calcification spots), ex vivo CT was assumed to benefit from higher resolution. Ex vivo ultrasound was used as the reference for applicable ultrasound features because of its dimensional advantages and unrestricted acoustic conditions. As in vivo ultrasound did not permit volumetric analysis, ex vivo ultrasound volumes were compared with ex vivo CT volumes.

All statistical analyses were performed on SPSS V25 (IBM, Armonk, NY, USA). Continuous parameters are reported as mean ± standard deviation or point estimate with 95% confidence intervals; categorical variables are presented as frequencies. Significance was defined as *p* ≤ 0.05; trends were defined as *p* ≤ 0.1.

## 3. Results

### 3.1. Computed Tomography

**Figure 2:**
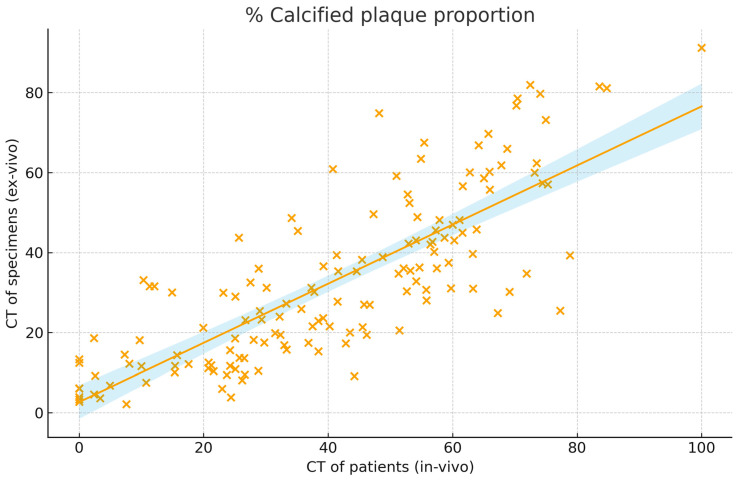
Correlation of calcified plaque components on computed tomography (CT) of patients and plaque specimens (n = 152).

**Figure 3:**
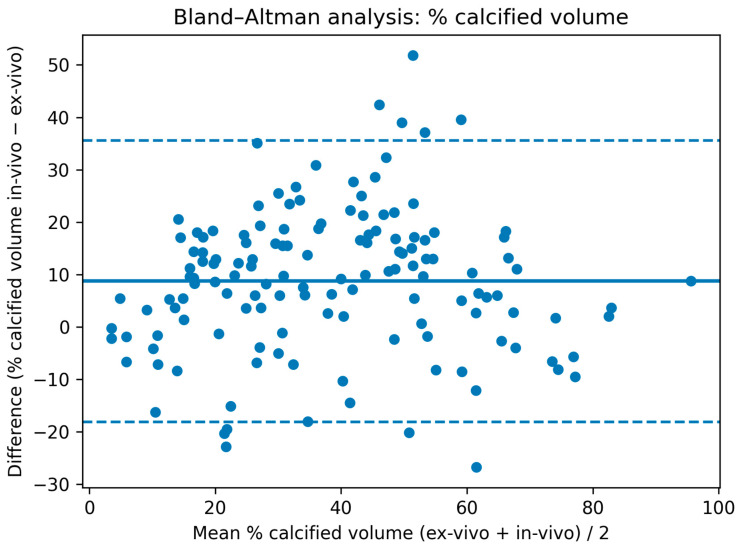
Bland–Altman analysis for the % calcified volume on in and ex vivo computed tomography.

CT of patients and ex vivo CT (Table 1) showed moderate to strong correlation for total, calcified, and non-calcified plaque volumes (r = 0.55 to 0.79; CCC = 0.43 to 0.74), with the highest correlations observed for relative calcified and soft plaque volumes (r = 0.79 (0.72–0.84), CCC = 0.74 (0.67–0.79)) (Figure 2). Mean HU values of the entire plaque also correlated well (r = 0.79 (0.72–0.84), CCC = 0.40 (0.37–0.43)). HU metrics of the calcified component showed moderate correlation (r = 0.53 (0.40–0.64), CCC = 0.13 (0.10–0.16)). On Bland–Altman analysis in vivo CT slightly overestimated the % calcified volume (mean bias: 8.7%) with moderate limits of agreement (95% limits of agreement: −18.1 to 35.5%) (Figure 3).

Agatston and calcium scores demonstrated strong correlation despite differing calculation algorithms (r = 0.78 to 0.80; CCC = 0.63 to 0.76). In contrast, most non-calcified plaque parameters showed negligible to weak correlation between modalities (r = −0.01 to 0.15; CCC = 0.00 to 0.07), except for mean HU values with higher correlation (r = 0.34 (0.19–0.47), CCC = 0.11 (0.06–0.15)).

The coefficient of variation showed weak correlation for the calcified component (r = 0.31 (0.16–0.45), CCC = 0.12 (0.06–0.17)) and weaker correlation for the entire or non-calcified plaque (r: 0.15 to 0.16, CCC: 0.07). Shares of cross-sections with profoundly localized calcification displayed weak correlation (r = 0.33 (0.18–0.46), CCC = 0.32 (0.17–0.45)). The number of calcified spots <1 mm also correlated weakly (r = 0.30 (0.15–0.44), CCC = 0.15 (0.07–0.21)).

The % calcified plaque proportion represents the share of the plaque volumes that is calcified with a Hounsfield Unit >130. The correlation is presented with a trend line and blue confidence margins and was strong at r = 0.79 (0.72–0.84). x spots represent the relative calcified volume as percentage of the whole plaque from ex vivo and in vivo images.

In vivo and ex vivo computed tomography measurements of the % calcified plaque volume were performed. In vivo CT overestimated calcified volume slightly as visualized in Figure 3 (bias: thick blue line = +8.7%) and 95% limits of agreement are moderate (dotted blue lines = −18.1 GS–35.5%).

### 3.2. Ultrasound

**Figure 4:**
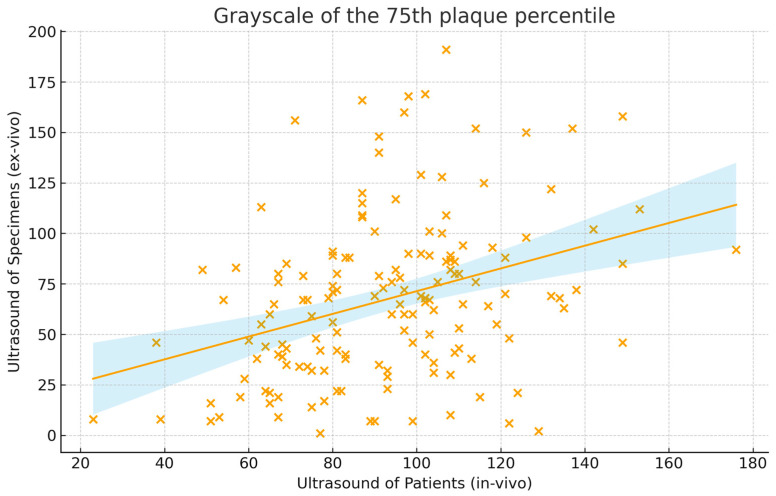
Correlation of ultrasound grayscale values from patients and plaque specimens (n = 156).

**Figure 5:**
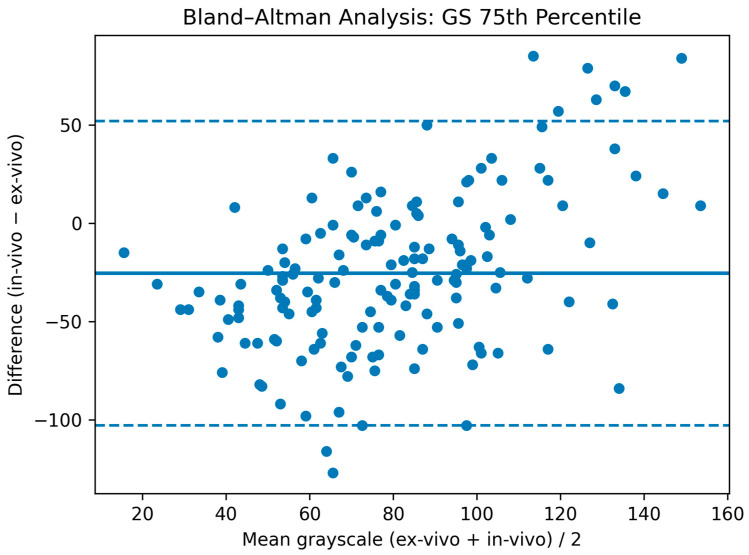
Bland–Altman analysis for the GS of the 75th plaque percentile on in and ex vivo ultrasound.

Overall, correlations between ultrasound of patients and ex vivo imaging were substantially weaker than those observed for CT, despite selecting representative slices from the entire plaque volume (Table 2). GS values showed weak correlation (GS average: r = 0.33 (0.18–0.46), CCC = 0.25 (0.14–0.36); GSM: r = 0.19 (0.03–0.34), CCC = 0.25 (0.14–0.35)). The GS of the 75th percentile of the plaque displayed the highest correlation, although remaining weak (r = 0.35 (0.20–0.48), CCC = 0.24 (0.14–0.34)) (Figure 4). The coefficient of variation correlated weakly (r = 0.20 (0.04–0.35), CCC = 0.11 (0.02–0.19). Plaque proportions of GS < 25 or <35 showed negligible correlation (r = 0.05, CCC = 0.02). On Bland–Altman analysis in vivo US systematically underestimated the GS of the 75th plaque quantile (mean bias: −25.5 GS) with wide limits of agreement (95% limits of agreement: −102.8 GS to 51.8 GS) (Figure 5).

The visual differentiation of plaque echogenicity in four categories showed weak to negligible correlation (Φ: 0.08–0.15) and binary assessments correlated weakly on juxtaluminal echolucency (Φ = 0.20 (0.04–0.35)) and the presence of DWA (Φ = 0.17 (0.01–0.32)).

Ex vivo plaque volumes derived from ex vivo CT and ex vivo ultrasound correlated moderately (CT: 551.2 ± 296.1 mm^3^; US: 538.7 ± 154.1 mm^3^; r = 0.59 (0.48–0.68), CCC = 0.48 (0.39–0.56)). Ultrasound underestimated plaque volume due to acoustic shadowing.

The grayscale of the 75th plaque percentile represents the grayscale under which 75% of the plaque is situated. The correlation is presented with a trend line and blue confidence margins and was weak at r = 0.35 (0.20–0.48). x spots represent the normalized grayscale of ex vivo and in vivo at the 75th plaque percentile.

In vivo and ex vivo ultrasound measurements of the grayscale of the 75th plaque quantile (i.e., the grayscale that is higher than that of 75% of the plaque) were performed. In vivo ultrasound underestimated GS as visualized in Figure 5 (bias: thick blue line = −25.5 GS) and 95% limits of agreement are wide (dotted blue lines = −102.8 GS–51.8 GS).

## 4. Discussion

The clinical relevance of ex vivo versus in vivo imaging depends largely on technical comparability, acquisition settings, and dimensional differences between modalities. When comparable equipment and standardized phantoms are used, ex vivo and in vivo CT can achieve high agreement, but ultrasound shows poor correlation. On CT, plaque proportions—such as calcified and non-calcified proportions—show the highest correlation, while heterogeneity metrics only correlate moderately. On US, objective grayscale metrics displayed higher, but still weak correlation and visual assessments were correlated in a weak to negligible manner. The inclusion into machine learning algorithms from ex vivo studies appears reasonable, but only for CT calcification analyses.

### 4.1. Ultrasound: Dimensional Differences and the Need for Volumetric Ultrasound in Patients

Ex vivo and in vivo ultrasound were acquired on the same equipment and are technically well matched. Nonetheless, dimensional discrepancies between in vivo and ex vivo imaging markedly limit direct comparability. Ex vivo US volumes showed moderate correlation with ex vivo CT (r = 0.59 (0.48–0.68)), reflecting uncertainties in measurement, voxel conversion, and acoustic shadow-related volume loss. Although GSM ex vivo appears sufficiently accurate to detect lipid-rich and fibrous plaque, these findings should not influence machine learning algorithms, as they cannot be sufficiently translated to an in vivo setting [9,11]. Whether even more sophisticated techniques can be translated to in vivo remains questionable, as these are even more influenced by effects of surrounding tissue [12,13]. Even when experienced sonographers select the “most representative” image in patients, correlations with volumetric ex vivo metrics remained weak, with the only slight correlation appearing concerning the objective predominant plaque component (75th percentile of GS). Underestimation of GS values by in vivo ultrasound further highlights the main limitation of acoustic shadowing which artificially lowers GS values across the entire plaque when not assessing the lesion volumetrically.

Diffuse features such as discrete white areas (DWA) correlated poorly. These findings suggest that 2D ultrasound inherently fails at complex spatial plaque characteristics, underscoring the potential benefit of 3D in vivo ultrasound acquisition for future clinical practice, but also the limited translational potential of ex vivo ultrasound for machine learning models [25].

Binary features such as echogenicity showed limited agreement; however, clinical ultrasound typically integrates multiple planes rather than relying on a single slice as in our study, which may explain some of the discrepancy. Potential observer bias may have contributed. Since sonographers cannot always be blinded to symptoms, more vulnerable appearing cross-sections may have been chosen in symptomatic patients. This highlights how objective ex vivo imaging provides valuable reference data for improving quantitative ultrasound assessment and minimizing bias.

### 4.2. CT: High Agreement and Remaining Limitations

Ex vivo CT was performed on a clinical scanner, enabling high-resolution settings to be translated into in vivo use, albeit with radiation dose considerations. Ex vivo conditions provide ideal contrast, absence of patient motion, and maximal HU separation due to the air–tissue interface. By contrast, in vivo scans often use contrast medium, which introduces pharmacologic risk and alters HU relationships, though modern reconstruction algorithms increasingly mitigate these effects [26].

Despite substantial methodological differences, CT demonstrated strong cross-modality correlation, particularly for density metrics, calcification scores, and relative volumes (e.g., *r* up to 0.80 for calcium and Agatston scores). This aligns well with previous publications in smaller samples, however only for the whole and calcified plaque [8,9,18]. In contrast to a previous study, we found overestimation of calcified volume instead of underestimation by in vivo CT, potentially due to partial volume effects from contrast agents that need to be considered for translational considerations [18]. These findings suggest that quantitative CT biomarkers are broadly transferable between ex vivo and in vivo imaging, especially when in vivo plaque volume is approximated using multi-slice rather than single-slice measurements.

Non-calcified plaque parameters correlated less strongly, likely due to inferior in vivo resolution and partial-volume effects. Similarly, spatially dependent metrics such as profound calcification were likely influenced by methodologic differences in slice selection. In vivo underdetection of calcified spots emphasizes limitations of conventional CT resolution and highlights the relevance of modern high-resolution acquisitions and possibly non-contrast CT for plaque calcium characterization.

Heterogeneity metrics demonstrated only limited agreement, possibly reflecting combined effects of reconstruction kernels, voxel sampling differences, and HU scaling. Because heterogeneity indices should theoretically be robust to such differences, dedicated in vivo validation with harmonized acquisition settings remains necessary [19].

### 4.3. Artificial Intelligence: Translational Potential of Quantitative Plaque Features Ex Vivo or In Vivo

The quantitative imaging features tested for ex vivo to in vivo correlation in this study—particularly relative volumes, HU-densities, and calcification characteristics—provide potential inputs well-suited for AI-based risk prediction models, even when primarily examined ex vivo. Especially for CT metrics, which appear most relevant to these prediction models, correlation was sufficiently high with acceptable agreement. Similar to previous work showing that low calcified volume and high non-calcified plaque heterogeneity predicts neurologic symptoms independently of stenosis and cardiovascular risk factors, our findings confirm that clinically accessible imaging can capture biologically meaningful plaque vulnerability features [15].

Such metrics constitute key building blocks for machine learning and deep learning frameworks. Recent advances demonstrate that AI can extract spatial patterns from medical images with high predictive value [6,7]. Convolutional neural networks can identify subtle patterns in plaque composition and distribution, while multimodal fusion models can integrate complementary data from ultrasound and CT to generate individualized stroke-risk predictions. While our data do not implement or analyze these approaches, such promising techniques may benefit from utilizing high-resolution imaging, only applicable ex vivo for CT, and question the consideration of ex vivo ultrasound features. These approaches have the potential to outperform traditional stenosis-based risk stratification and assist clinicians in identifying patients who would derive the greatest benefit from revascularization or intensified medical therapy. Especially plaque components, calcification metrics, and indices may be transferable from ex vivo to in vivo for these models.

Future studies may evaluate AI models using plaque features, even when generated ex vivo under aforementioned circumstances, validate performance across imaging modalities, and explore clinical deployment. Ultimately, AI offers a promising framework to achieve translation from quantitative imaging features to personalized clinical decision-making.

## 5. Limitations

First, clinical in vivo ultrasound is routinely acquired as two-dimensional images, whereas ex vivo ultrasound enables volumetric imaging. Despite careful selection of the most representative plaque image by experienced observers, this limits comparability and may contribute to the weaker correlation. This further highlights the restricted translational potential of ex vivo ultrasound features. Second, ex vivo CT was performed under idealized conditions without limitations to acquisition time, patient motion, radiation exposure, and air-tissue interfaces. Third, formalin fixation of specimens can induce tissue shrinkage and alter attenuation properties and may have caused discrepancies between in vivo and ex vivo imaging. Fourth, in vivo ultrasound measurements might be subject to interobserver variability. Fifth, we did not perform histology as reference standard for plaque characteristics. Sixth, the study was conducted at a single tertiary cardiovascular center, which may limit generalizability. Finally, this study discusses implications for radiomics and artificial intelligence research but did not validate a predictive AI model and no external validation was performed. The present analysis should therefore be interpreted as an assessment of feature translatability.

## 6. Conclusions

Across modalities, the strongest agreement between ex vivo and in vivo imaging was achieved when acquisition settings, dimensionality, and measurement methodology were most comparable. Under such conditions, ex vivo imaging can provide meaningful translational insights, validate clinical imaging biomarkers, and support the development of advanced analytical models, including AI models. Integrating quantitative features, such as calcification proportion or calcification scores, into machine learning frameworks represents a promising next step to improve individualized stroke-risk stratification and optimize treatment decisions.

## Figures and Tables

**Table 1:** Comparative analysis of patients and plaque specimens on computed tomography.

Parameter (Average ± SD)	Plaque	Patient	r ^a^ (CI ^b^)	CCC ^c^ (CI)
Plaque volume (mm^3^)	528 ± 292	754 ± 418	0.55 (0.43–0.65)	0.43 (0.34–0.51)
Calcified volume (%)	32.5 ± 20.7	40.3 ± 22.2	0.79 (0.72–0.84)	0.74 (0.67–0.79)
Non-calcified volume (%)	67.5 ± 20.7	59.7 ± 22.2	0.79 (0.72–0.84)	0.74 (0.67–0.79)
Whole plaque average (HU ^d^)	456 ± 373	205 ± 154	0.79 (0.72–0.84)	0.40 (0.37–0.43)
Whole plaque CV ^e^	1.6 ± 1.7	0.78 ± 0.51	0.16 (0.0–0.31)	0.07 (0.0–0.14)
Calcified plaque average (HU)	902 ± 284	357 ± 182	0.53 (0.40–0.64)	0.13 (0.1–0.16)
Calcified plaque CV	0.88 ± 0.50	0.48 ± 0.17	0.31 (0.16–0.45)	0.12 (0.06–0.17)
Non-calcified plaque average (HU)	59 ± 36.4	49 ± 35.0	0.34 (0.19–0.47)	0.11 (0.06–0.15)
Non-calcified plaque CV	0.82 ± 0.84	0.30 ± 0.32	0.15 (−0.01–0.31)	0.07 (0.04–0.10)
Agatston score (Pixel × HU × 1000)	136 ± 145	126 ± 118	0.78 (0.71–0.84)	0.76 (0.69–0.82)
Calcification score (mm × HU × 1000)	32 ± 28	19 ± 18	0.80 (0.73–0.85)	0.63 (0.58–0.67)
Profoundly localized calcification (%)	37 ± 39	29 ± 46	0.33 (0.18–0.46)	0.32 (0.17–0.45)
Calcification spots <1 mm (n)	6 ± 5	3 ± 2	0.30 (0.15–0.44)	0.15 (0.07–0.21)

a: Pearson’s correlation coefficient, b: 95% confidence interval, c: Lin’s concordance correlation coefficient, d: Hounsfield unit, e: coefficient of variation.

**Table 2:** Comparative analysis of patients and plaque specimens on ultrasound.

**Parameter (Average ± SD)**	**Plaque**	**Patient**	**r ^a^ (CI ^b^)**	**CCC ^c^ (CI)**
Plaque volume (mm^3^)	539 ± 154	551 ± 296 (CT ^d^)	0.59 (0.48–0.68)	0.48 (0.39–0.56)
Average (GS ^e^)	61 ± 16	50 ± 27	0.33 (0.18–0.46)	0.25 (0.14–0.36)
Median GSM (GS)	40 ± 15.5	40 ± 30	0.19 (0.03–0.34)	0.16 (0.03–0.28)
GS of the 75th quantile (GS)	92 ± 25	67 ± 38	0.35 (0.20–0.48)	0.24 (0.14–0.34)
CV ^f^	0.65 ± 0.22	0.96 ± 0.50	0.20 (0.04–0.35)	0.11 (0.02–0.19)
Proportion with GS < 25 (%)	41 ± 8	42 ± 31	0.05 (−0.22–0.32)	0.02 (−0.11–0.15)
Proportion with GS < 35 (%)	49 ± 8	54 ± 47	0.05 (−0.22–0.32)	0.02 (−0.07–0.11)
**Binary Parameter (n (%))**	**Plaque**	**Patient**	**Phi Correlation Coefficient (** **Φ)**	
Echolucent	31 (20)	17 (11)	0.14 (−0.03–0.32)	
More echolucent	37 (24)	29 (19)	0.08 (−0.18–0.32)	
More echogenic	54 (35)	52 (33)	0.15 (−0.01–0.31)	
Echogenic	37 (24)	59 (38)	0.10 (−0.12–0.32)	
Juxtaluminal echolucency	38 (24)	16 (10)	0.20 (0.04–0.35)	
DWA ^g^	94 (60)	29 (19)	0.17 (0.01–0.32)	

a: Pearson’s correlation coefficient, b: 95% confidence interval, c: Lin’s concordance correlation coefficient, d: assessed on ex vivo computed tomography, e: grayscale, f: coefficient of variation, g: discrete white areas.

## Data Availability

The data underlying this article are summarized in the article; detailed data (patient by patient) are available upon specific request to the corresponding author.

## Abbreviations

The following abbreviations are used in this manuscript:

AI	Artificial Intelligence
CCC	Lin’s Concordance Correlation Coefficient
CEA	Carotid Endarterectomy
CT	Computed Tomography
CV	Coefficient of Variation
DWA	Discrete White Areas
GS	Grayscale
GSM	Grayscale Median
HU	Hounsfield Unit
MRI	Magnetic Resonance Imaging
Phi (Φ)	Phi Correlation Coefficient
r	Pearson’s Correlation Coefficient
US	Ultrasound
